# Harnessing chemical pressure

**DOI:** 10.1093/nsr/nwad234

**Published:** 2023-09-07

**Authors:** Ho-kwang Mao

**Affiliations:** Shanghai Advanced Research in Physical Sciences (SHARPS), China

Pressure profoundly changes all physical characteristics of a condensed matter and provides a vast and fertile dimension to search for materials with extremely favorable properties for technological applications [1,2]. However, these favorable properties are only optimally tuned at a specific high pressure and will vanish or revert at the ambient pressure, rendering them merely of academic interest.

A promising solution is to take advantage of the ‘chemical pressure’. When a relatively large atom is replaced by a smaller atom, it will be locally ‘squeezed’ by the surrounding crystal lattice with an additional chemical pressure, *P*_chem_, and exhibits pressure-induced functional changes even at ambient pressure. Using this chemical pressure to achieve desired properties has been a long-standing idea. For instance, the concept of chemical pressure inspired the replacement of La in the 1987 Nobel Prize-winning lanthanum barium cuprite [3] that reached 30 K superconducting Tc, with Y [4] that reached an astounding Tc of 93 K and kicked off the era of the YBCO superconductor revolution. The discovery of hydrogen-dominant hydrides as near room Tc superconductors [5,6] was initially an attempt to ‘precompress’ hydrogen with chemical pressure [7] for metallization of hydrogen. Regardless of these impressive successes, however, fundamental questions remain. How do we measure the chemical pressure localized at a specific atom? Can it be sufficiently large to create metallic hydrogen or to stabilize exotic functional materials for applications under ambient conditions?

**Figure 1.**
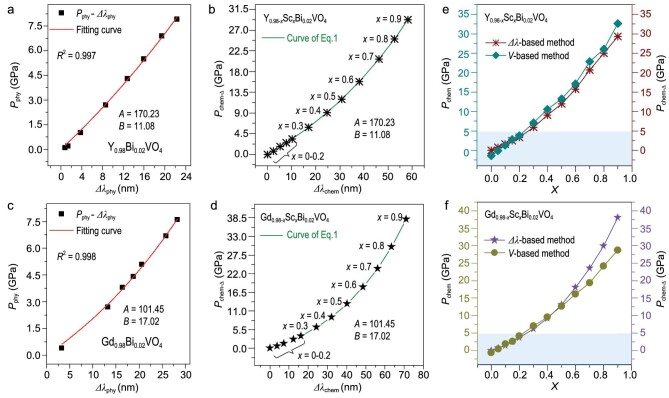
(a) Pressure-dependent spectra shift (*Δλ*_phy_) of Y_0.98_Bi_0.02_VO_4_ and (c) Gd_0.98_Bi_0.02_VO_4_ fitted by the Mao–Bell equation [9]. (b) The fitted curve also reflects the relationship between the spectra shift (*Δλ*_chem_) and (d) the local chemical pressure (*P*_chem_) of Y_0.98__–_*_x_*Sc*_x_*Bi_0.02_VO_4_ and Gd_0.98__–_*_x_*Sc*_x_*Bi_0.02_VO_4_. The comparison of relationships between *P*_chem_ and *x* are determined by using *V-* and *λ-*based methods for (e) Y_0.98__–_*_x_*Sc*_x_*Bi_0.02_VO_4_ and (f) Gd_0.98__–_*_x_*Sc*_x_*Bi_0.02_VO_4_. Reproduced from Ref. [8].

To address these questions, Zhou *et al.* [8] designed an innovative study on pressure-tuned luminescence of Bi^3+^ dopant in yttrium and gadolinium orthovanadates with a general formula of AVO_4_ where the dodecahedral A sites are filled with various large cations, Y^3+^ and Gd^3+^, as well as the dopant Bi^3+^. The Bi^3+^ emission spectra strongly depend upon the A site volume, which can be compressed by application of external pressure *P*_phy_ or by varying the chemical substitutions of Y, Gd and Sc of different ionic radii to generate local chemical pressure *P*_chem_. The multivariable experiments include measurements of the AO_8_ polyhedral volume (*V*) and the Bi^3+^ emission peak wavelength (*λ*) as functions of external pressure (*P*_phy_) and chemical substitution (*x*) of Y_0.98__–_*_x_*Sc*_x_*Bi_0.02_VO_4_ and Gd_0.98__–_*_x_*Sc*_x_*Bi_0.02_VO_4_ that generates the chemical pressure. The comprehensive approach first calibrated *λ* as a function of *P*_phy_ without *P*_chem_ (*x* = 0) for yttrium orthovanadate and gadolinium orthovanadate (Fig. 1a and c). Then the *λ*–*P* calibration is used to determine *P*_chem_ as a function of *x* for *P*_phy_ = 0 (Fig. 1b and d). The *P*_chem_ value calculated on the basis of the *λ*–*P* calibration agrees very well with that on the basis of the *V*–*P* relations (Fig. 1e and f).

The study [8] provides valuable answers to the aforementioned questions. The functional effect induced by chemical pressure on localized atoms, such as luminescence, is indeed equivalent to that induced by the physical pressure on the bulk crystal and can be used to quantify the chemical pressure. The finding of chemical pressure as big as 30–40 GP offers hope for stabilizing hydrogen-dominant high Tc superconductors. Recent efforts on lowering the synthesis pressure to <100 GPa with ternary hydrides [6] are promising with the additional chemical pressures.
